# Monitoring *Spongospora subterranea* Development in Potato Roots Reveals Distinct Infection Patterns and Enables Efficient Assessment of Disease Control Methods

**DOI:** 10.1371/journal.pone.0137647

**Published:** 2015-09-09

**Authors:** Tamilarasan Thangavel, Robert S. Tegg, Calum R. Wilson

**Affiliations:** Tasmanian Institute of Agriculture, School of Land and Food, University of Tasmania, New Town Research Laboratories, 13 St John’s Avenue, Tasmania, 7008, Australia; Università Politecnica delle Marche, ITALY

## Abstract

*Spongospora subterranea* is responsible for significant potato root and tuber disease globally. Study of this obligate (non-culturable) pathogen that infects below-ground plant parts is technically difficult. The capacity to measure the dynamics and patterns of root infections can greatly assist in determining the efficacy of control treatments on disease progression. This study used qPCR and histological analysis in time-course experiments to measure temporal patterns of pathogen multiplication and disease development in potato (and tomato) roots and tubers. Effects of delayed initiation of infection and fungicidal seed tuber and soil treatments were assessed. This study found roots at all plant developmental ages were susceptible to infection but that delaying infection significantly reduced pathogen content and resultant disease at final harvest. The pathogen was first detected in roots 15–20 days after inoculation (DAI) and the presence of zoosporangia noted 15–45 DAI. Following initial infection pathogen content in roots increased at a similar rate regardless of plant age at inoculation. All fungicide treatments (except soil-applied mancozeb which had a variable response) suppressed pathogen multiplication and root and tuber disease. In contrast to delayed inoculation, the fungicide treatments slowed disease progress (rate) rather than delaying onset of infection. Trials under suboptimal temperatures for disease expression provided valuable data on root infection rate, demonstrating the robustness of monitoring root infection. These results provide an early measure of the efficacy of control treatments and indicate two possible patterns of disease suppression by either delayed initiation of infection which then proceeds at a similar rate or diminished epidemic rate.

## Introduction

*Spongospora subterranea* f.sp. *subterranea* is a soil-dwelling obligate cercozoan plant pathogen that invades potato roots, stolons and tubers, reducing root function [1,2] and causing the blemish disease powdery scab in tubers [3]. Losses to the Australian potato processing industries are estimated at A$13.4M per annum [4]. *S*. *subterranea* is also the vector of *Potato mop top virus*, a significant disease in many parts of the world [3], and may provide an entry point for other root and tuber-invading pathogens [3,5]. There are currently no sustainable and reliable controls for the pathogen [6].

The pathogen survives for prolonged periods in the soil and on seed tubers as conglomerates of resting spores (sporosori) from which motile primary zoospores are released. These actively swim toward host roots, encyst on the root surface and transfer the contents of the zoospore within root cells [1,6]. Root infection progresses with the formation of a plasmodium which becomes a sporangium and produces secondary zoospores that are released from the cell and initiate new infections within the root system and newly developing tubers in a polycyclic manner [1]. As the disease progresses there is a change from the zoosporangial to the sporogenic (resting spore) stage of the life cycle with formation of root and tuber galls where resting spores are produced within sporosori [1,7]. These are released into the soil providing new sources of inoculum.

As an obligate pathogen with infections occurring beneath the soil, the study of disease epidemics is technically difficult. Recent advances in pathogen detection and quantitation using qPCR have enabled researchers to measure the pathogen during root infection and assessments of cultivar susceptibility made [8]. Previous research has identified that the critical period for tuber infection is shortly after tuber initiation where the host cells are susceptible to pathogen penetration [9]. In controlled experiments where application of *S*. *subterranea* inoculum could be delayed, tuber symptoms were greatest when inoculum was applied early at tuber set, compared to later applications [10,11]. However, these experiments examined tuber disease only and did not assess root infection and root galling. Until this present study it was unknown whether a similar defined period of susceptibility existed in potato roots and what affect a delay in inoculum application would have on the dynamics of root infection.

In New Zealand and Europe a range of fungicides applied as to seed tubers or the soil at planting have successfully reduced the incidence of powdery scab increasing the yield of marketable tubers [12,13]. While, such fungicides may be an important part of an integrated management strategy for this disease [14], their impact on root infection and root disease has not been examined. Recent evidence suggests early associations between pathogen and the root are critical to subsequent disease expression [8]. Therefore, practices or strategies that can delay this interaction or slow subsequent disease progress may provide viable control options.

The aims of this study are to (1) develop a system for measuring temporal patterns of pathogen replication and disease within potato roots, and use these data to (2) identify whether potato roots, like tubers, have distinct periods of susceptibility to *S*. *subterranea* infection, (3) determine whether the epidemic rate within roots changes with respect to plant age at onset of infection, and (4) assess the effect of seed-tuber and soil applied fungicides on pathogen infection rate and epidemic patterns. We hypothesise that monitoring temporal root infection patterns can provide a reliable assessment of the ability of control strategies to mitigate root and tuber disease.

## Materials and Methods

### Ethics statement

No specific permissions were required for these pot and field trials. The studies did not involve endangered or protected species. Five pot trials were conducted in New Town (southern Tasmania, 147°17’57.21”E, 42°51’24.55”S), and two field trials in Wesley Vale (north-west Tasmania, 146° 24’ 41”E, 41° 11’ 38”S) over the period 2012–2014.

### Impact of delayed *S*. *subterranea* infection on pathogen replication and disease

Three pot trials (PT1, 2 and 3) examined the impact of delaying inoculation of *S*. *subterranea* on root infection and root and tuber disease in potato (PT1 and 2) and on root infection in tomato (PT3). Tomato was used to enable experimental effects to be tested across more than one species as it is a known susceptible host of the pathogen and provides a convenient model plant system. All trials occurred within a glasshouse environment with temperatures maintained at 16–22°C (PT1) or 16–35°C (PT2 and 3). Plastic pots (20 cm diameter, 4.5 L volume), were filled with pasteurized potting mix containing sand, peat, and composted pine bark (10: 10: 80; pH 6.0) and premixed with Osmocote 16–3.5–10 NPK resin coated fertiliser (Scotts Australia Pty Ltd.) at the rate of 6 kg/m3. In PT1 (11 August 2011—winter) and PT2 (8 January 2012—summer) pathogen-free mini-tubers of potato cultivars Russet Burbank and Desiree were planted at 10 cm depth (one tuber per pot) and in PT3 (22 January 2012—summer), 2-week-old healthy tomato seedlings of cv. Mortgage and Roma were transplanted into pots (one plant per pot).

*S*. *subterranea* inoculum was obtained from heavily diseased potato tubers that had been stored (for a maximum of three months) in ambient cool conditions (10°C) until use. Inoculum was prepared using a modification of previous methods [15,16]. Peel (to a depth of 1 cm) was removed from infected tubers, air-dried at 25°C, ground and sieved (35 μm); the resultant inoculum was stored at 4°C. Three days prior to application, inoculum powder was incubated in nutrient solution [17] for 3 days at 18–20°C in the dark. Ten mL of inoculum solution (adjusted to ~50 sporosori/10 μl using a haemocytometer i.e. 50,000 sporosori/pot) was applied to the soil surface of the pots [15]. In PT1 and PT2 pots were amended with *S*. *subterranea* inoculum at six different stages of plant development: emergence, 10 days after emergence (DAE), 20 DAE, 30 DAE, 40 DAE and 50 DAE. PT1 included an additional inoculum treatment at 60 DAE. Plants from individual replicate pots were destructively harvested at 15 days intervals for up to five harvest periods after initial inoculum treatment and root and tuber tissues sampled for further analysis. In PT3, pots were amended with *S*. *subterranea* inoculum at four different stages: transplanting, 20 days after transplanting (DAT), 40 DAT and 60 DAT. Plants were destructively harvested at 15 day intervals for up to seven harvest periods after initial inoculum treatment. For all PTs individual treatment combinations were replicated three times with pots arranged in a randomised complete block design and hand watered (when required) to maintain constant wet soil conditions. There were no pesticides or additional fertilizer applications. Harvested roots and tubers were assessed for disease in PT1 and PT2 while pathogen content in roots was quantified by qPCR in PT1-3.

### Impact of seed and soil applied fungicides on pathogen replication and disease

#### Pot trials

Two pot trials (PT4, PT5) examined the impact of mancozeb, applied as a soil treatment, on potato plants grown in potting soil inundated with *S*. *subterranea*. All trials were conducted in an ambient, outdoor environment in New Town, Tasmania. PT4 was grown over summer (mean minimum of 12°C and mean maximum of 22°C) and PT5 over autumn/winter (mean minimum and maximum temperatures of 7 and 17°C). In PT4 disease-free mini-tubers of Russet Burbank were used and *S*. *subterranea*-infested field soil incorporated through the potting mix 1 week prior to planting, providing a high inoculum pressure (~ 40 ng *S*. *subterranea* DNA/g soil). Treatments included mancozeb (7.5 kg/ha i.e. 18L/ha in 1000 L of water) sprayed into a 10 cm-deep furrow in individual pots, just prior to planting and a water spray control. A control treatment without *S*. *subterranea* inoculum or fungicide was also included. Five tubers were planted at 10 cm depth (3 January 2012) in individual pots (20 cm diameter, 4.5 L volume) with six destructive harvests; 15, 30, 45, 60 and 75 DAE (root assessment), and after senescence (tuber assessment). There were three replicates for each harvest. In PT5 two-week old disease-free tissue cultured plantlets of Russet Burbank and Desiree were used with inoculum prepared as for PT1-3 trials and applied to potting soil just prior to planting. Treatments included three rates of mancozeb (equivalent to 3.25, 7.5, 15.0 kg/ha) sprayed into a 5cm-deep furrow just prior to planting and a water spray control. A control with no inoculum or fungicide was included. Seedlings were transplanted (4 April 2012) into individual pots (20 cm diameter, 4.5 L volume) with plants destructively harvested at 15 days intervals. In both trials, each treatment was replicated three times (one plant per pot) with pots arranged in a randomised complete block design and hand watered (when required) to maintain constant wet soil conditions. There were no additional pesticide or fertilizer applications. In PT4 pathogen content in roots by qPCR was quantified as well as root and tuber disease. In PT5 only root disease (zoosporangial score) was measured.

#### Field trials

Two field trials were established (12 October 2013) on commercial farms at Wesley Vale NW Tasmania (climate high rainfall season with mean minimum temperatures of 10°C and mean maximum temperatures of 20°C) on potato growing soils typical of the region; field trial 1 (FT1) on a red ferrosol soil and field trial 2 (FT2) on a brown dermosol soil. Powdery scab had been recorded in recent potato crops at both sites. Analysis of soil sampled just prior to planting by qPCR [18,19] gave very low *S*. *subterranea* inoculum levels of 415 and 33 pg DNA/g soil for F1 and F2 respectively. Planting material for all field trials was commercial seed of both ‘Russet Burbank’ and ‘Innovator’ with visually obvious powdery scab symptoms; each individual seed tuber having a surface cover score of 2 (surface cover percentage of *c*. 5–10% powdery scab) which corresponded to a qPCR in the tuber peel of ~100 ng *S*. *subterranea* DNA/g sample. Seed and soil chemical treatments were applied at planting. Treatments were seed-tubers dipped in (i) 1% formalin, (ii) fluazinam (7.5 g/10 kg seed) or (iii) mancozeb (32 g/10 kg seed), and soil in-furrow sprays of (iv) fluazinam (3.0 kg/ha i.e. 6L/ha in 1000 L of water) or (v) mancozeb (7.5 kg/ha i.e. 18L/ha in 1000 L of water) applied with a knap sack sprayer (Matabi 18L Elegance, Goizper Spraying, Portugal) and an untreated control.

Each treatment was replicated two times in each trial. Plots contained 15 seed tubers, spaced at 30 cm, with plots arranged in a randomized split-plot design. Fertiliser and irrigation scheduling followed standard commercial practice with no additional seed or soil pesticides applied. In both FTs the average emergence date was on 25^th^ November 2013 with destructive sequential harvesting of one plant per plot made at 15, 30, 45, 60 and 75 DAE. Plants were gently uprooted with roots kept for infection, gall assessment and pathogen quantification by qPCR (stored at 4°C for up to two days). All other plants (10 per plot) were grown until senescence, tubers harvested and a random sample of 50 tubers per plot selected for disease assessment.

### Pathogen DNA quantification

DNA was extracted and quantified from soil material and tuber skin (peel) using established protocols of the commercial Root Testing Service of the South Australian Research Development Institute, Adelaide, South Australia, Australia [18,19,20]. This was to confirm the presence and quantity of pathogen in soil and tuber samples. DNA extraction from root tissue followed a modified technique with established quantification methodologies [8,21]. Essentially, whole root samples were washed thoroughly in running tap water to remove soil particles and representative sub-samples were stored at -80°C. Samples were dried at 30°C for 2–3 days and ground using mortar and pestle. Ground samples (50 mg) were mixed with 50μl of nuclease-free water prior to DNA extraction. DNA was extracted using Power plant ®pro DNA isolation Kit with RNAase treatment (MO BIO Laboratories, Inc, Canada), with DNA yield quantified using the Qubit^®^ 2.0 Flurometer (Life technologies, Darmstadt, Germany). Primers for *S*. *subterranea* (SPO10, SPO11, probe) quantitation were from the ribosomal ITS region [8]. An internal control amplifying the conserved mitochondrial cytochrome oxidase (COX) gene from potato was run in all samples using previously reported primer (COX-F, COX-R) and probe (COX-P) sequences [21]. This was used as a positive internal control to confirm DNA quality, PCR amplification conditions and to normalise qPCR data for accurate quantification of the pathogen content.

Quantitative PCR was performed using 2 μl (10 ng/μl) of DNA template in 10 μl volume reactions using the Sensi FAST^TM^ Probe No-ROX Kit (Bioline Pty.Ltd, Australia) [8]. All amplifications were carried out in a Rotor Gene 6000 instrument (Corbett Life Science, Sydney, Australia) with a thermocycle of 95°C for 15 mins, then 40 cycles of 94°C for 15 s and 60°C for 60s, with three replicates per sample. Amounts of *S*. *subterranea* DNA in samples were calculated by an absolute quantification method, using a standard curve determined from the amplification of six ten-fold dilutions (1.3 μg to 13 pg) of a plasmid DNA containing the *S*. *subterranea* ITS gene kindly supplied by the South Australian Research and Development Institute, as previously described [8]. The efficiency of the standard curve derived from the plasmid dilutions was determined as 99%.

### Disease assessment

#### Root disease—root infection and galling

Root hair infection was assessed by microscopic examination using a method modified from Merz [17]. From each plant, three samples of root (2–5 cm long) were cut at *c*. 30–50 mm from the crown region and thoroughly washed. Specimens were mounted on a glass slide, stained with aqueous 0.1% (w/v) aniline blue for 5 mins, rinsed with water, and a drop of 50% (v/v) aqueous glycerol added. Samples were examined microscopically at 200x magnification (Leica DMLB, Type LB 30T compound microscope; Leica Microsystems). Fifteen fields of view were examined per slide with presence of zoosporangia rated: 0 = no zoosporangia; 1 = only a few zoosporangia on root hairs; 2 = 3–5 root hairs with zoosporangia; 3 = 6–10 root hairs with zoosporangia, moderate infection; 4 = >10 root hairs with zoosporangia, heavy infection [17].

A root galling score was given per plant based on a visual rating scale modified from van de Graff *et al*. [9]: of 0 = no galls; 1 = 1–2 galls; 2 = 3–10 galls mostly <2mm in diameter, 3 = more than 10 galls some of which are >2mm in diameter; 4 = most major roots with galls some or all >4mm in diameter.

#### Tuber disease—Powdery scab

Harvested tubers were stored at 4°C for up to 4 weeks, prior to disease assessment. Tubers were washed and each tuber (> 4 g) was scored for powdery scab severity according to a visual tuber surface cover score ranging from 0 to 6 (0 = no visible disease on tuber surface, 0.5 = ≤ 1%; 1 = ≥ 1–5%; 2 = ≥ 5–10%; 3 = ≥ 10–30%; 4 = ≥ 30–50%; 5 = ≥ 50–70%; 6 = ≥ 70% tuber surface affected). The percentage of tuber surface covered by lesions was then estimated by taking the mid values of these score ranges. The proportion of healthy tubers with no visible lesions was also recorded from which disease incidence was calculated [22].

### Data analysis

Data in all trials where time-course measurements were made (root DNA levels, root zoosporangia scores and root gall severity scores) were analysed for Area Under Disease Progress Curve (AUDPC) using the trapezoidal method—PROC ANOVA in SAS v9.3. All normalized DNA values were transformed to log (*Spongospora* DNA + 1) prior to AUDPC calculation. The mean AUDPC of each treatment were assessed by one-way analysis of variance (ANOVA) and using Tukey’s method significant results were grouped at 5% difference level (p = 0.05). Additionally, where different inoculation dates were utilised (PT 1–3) a modified AUDPC^i^ was also calculated measuring disease progress from the date of inoculum addition rather that the date of emergence. Data were truncated to ensure the same number of data points (three) was analysed for each curve. AUDPC^i^ were assessed by one-way ANOVA. Where parameters were discrete, single measurements (e.g. tuber disease) data was analysed by one-way ANOVA using GENSTAT (version 14.2). Data was only used where the assumptions of the general linear model could be met and where graphical diagnostics showed a normal distribution.

## Results

### Impact of delayed *S*. *subterranea* infection on pathogen replication and disease

#### Pathogen replication

**Fig 1:**
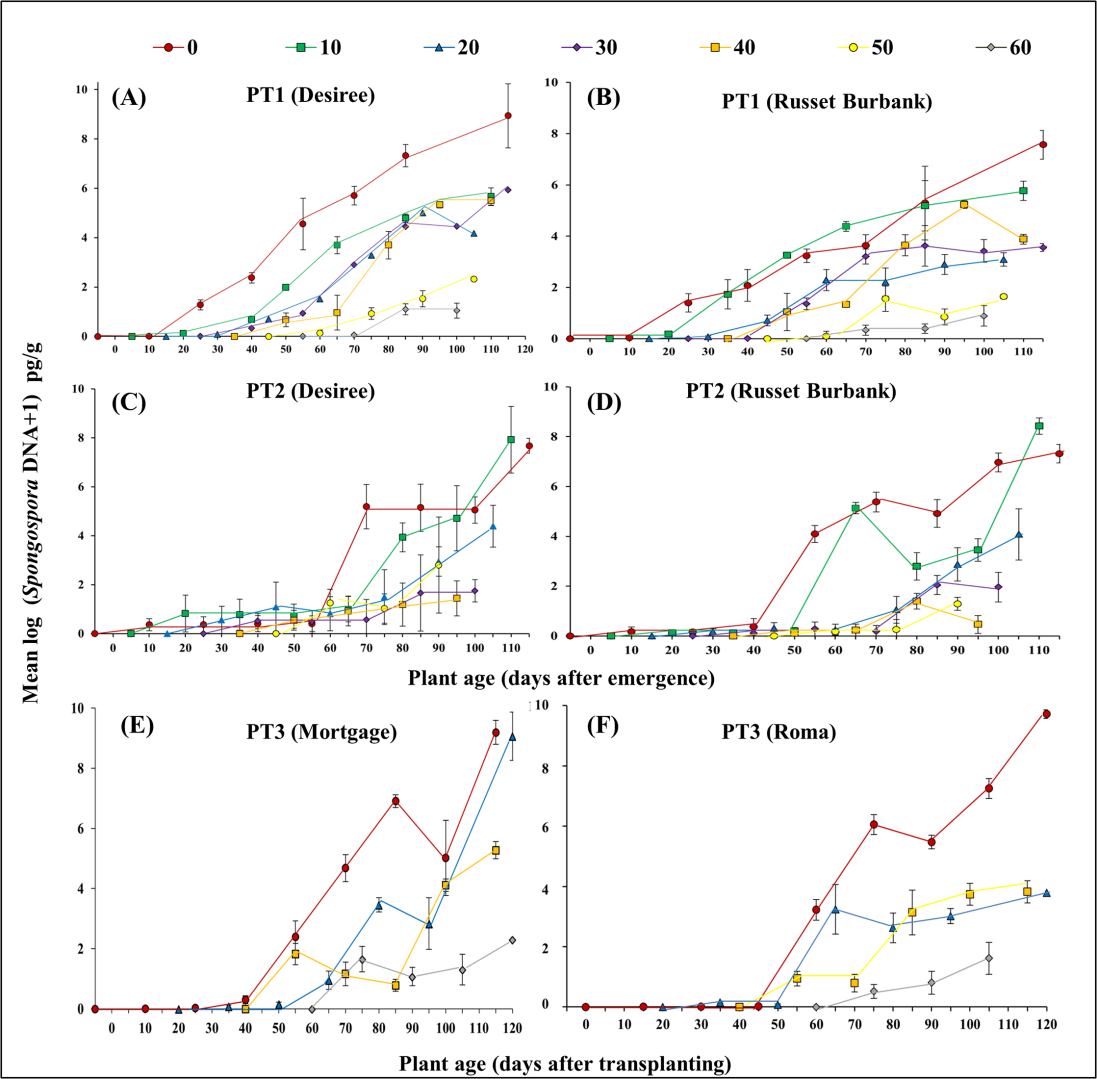
The effect of delayed inoculation on initiation of root infection and replication of *S*. *subterranea*. Results are for two potato cultivars in two pot trials (PT1—winter: A and B, PT2—summer: C and D) and in two tomato cultivars in one pot trial (PT3—summer: E and F). Markers represent the different inoculation treatment dates shown as days after emergence (DAE) or days after transplanting (DAT). Vertical bars are standard errors (n = 9).

In PT1, 2 and 3 levels of *S*. *subterranea* DNA within the root increased with time (Fig 1). As expected, where application of inoculum was delayed there was a delay in the onset of infection and a resultant decrease in the AUDPC. In PT1 inoculation at 50 and 60 DAE resulted in a lower AUDPC than inoculation at emergence, 10, 20, 30 and 40 DAE in both Desiree (p < 0.0001, Fig 1A) and Russet Burbank (p < 0.0001, Fig 1B) cultivars (Table 1A). Likewise in PT2 similar trends were observed with the early application at emergence producing significantly greater pathogen levels in the root than the delayed applications at 40 and 50 DAE for Desiree (p = 0.02, Fig 1C) and at 20, 30, 40 and 50 DAE for Russet Burbank (p = 0.001, Fig 1D; Table 1B). In PT 3, the earliest inoculation treatment at transplant produced a greater AUDPC than the three later inoculation treatments, 20, 40 and 60 DAT, in cv. Mortgage (p < 0.0001, Fig 1E) and cv. Roma (p < 0.0001, Fig 1F; Table 1B).

**Table 1:** Area Under Disease Progress Curve (AUDPC) for root pathogen and disease parameters following delayed inoculation (PT 1–3).

**A**												
	**PT1**											
**DAE**	**Pathogen DNA**				**Zoosporangial score**				**Root galling score**			
	**Desiree**		**Russet Burbank**		**Desiree**		**Russet Burbank**		**Desiree**		**Russet Burbank**	
	**AUDPC**	**AUDPC** ^**i**^	**AUDPC**	**AUDPC** ^**i**^	**AUDPC**	**AUDPC** ^**i**^	**AUDPC**	**AUDPC** ^**i**^	**AUDPC**	**AUDPC** ^**i**^	**AUDPC**	**AUDPC** ^**i**^
0	1168.6 ^a^	85.3	893.1 ^a^	84.6	25.0 ^a^	0.5	10	0.5	50.0 ^ab^	2.5	27.5 ^ab^	0
10	665.7 ^b^	60	794.4 ^a^	119.1	15.5 ^ab^	1.5	12.5	1	50.0 ^ab^	12.5	50.0 ^a^	5
20	435.9 ^b^	51.8	334.7 ^bc^	65.5	6.0 ^bc^	1.5	6	2	47.5 ^ab^	7.5	30.0 ^ab^	15
30	547.3 ^b^	87.8	462.6 ^b^	102.5	6.0 ^bc^	1.5	23	2	62.5 ^a^	7.5	30.0 ^ab^	15
40	451.1 ^b^	108.5	438.1 ^b^	127.3	5.0 ^bc^	1.5	6	2	37.5 ^abc^	7.5	25.0 ^ab^	15
50	126.6 ^c^	60.2	113.4 ^cd^	70.3	1.0 ^c^	1	2	2	10.0 ^bc^	7.5	15.0 ^ab^	15
60	56.8 ^c^	56.9	34.5 ^d^	34.5	0.0 ^c^	0	0	0	0.0 ^c^	0	0.0 ^b^	0
P	<0.0001	0.13	<0.0001	0.1	<0.0001	0.32	0.06	0.39	0.003	0.42	0.01	0.35
F value	69.32	2.48	46.43	2.89	13.1	1.17	2.75	1.13	5.91	1.08	4.16	1.22
**B**												
	**PT2**				**PT3**							
	**Desiree**		**Russet Burbank**		**Mortgage**		**Roma**					
	**AUDPC**	**AUDPC** ^**i**^	**AUDPC**	**AUDPC** ^**i**^	**AUDPC**	**AUDPC** ^**i**^	**AUDPC**	**AUDPC** ^**i**^				
0	840.6 ^a^	92.6	789.6 ^a^	75.2	827.6 ^a^	53.2	929.1 ^a^	56.6				
10	529.6 ^ab^	105.2	447.8 ^ab^	110.1	-	-	-	-				
20	304.4 ^ab^	86.8	227.8 ^b^	82.2	557.8 ^b^	99.3	455.0 ^b^	162.2				
30	132.5 ^ab^	85.5	94.8 ^b^	75.3	-	-	-					
40	107.3 ^b^	75.2	52.0 ^b^	45.2	474.8 ^b^	189.9	439.3 ^b^	216.9				
50	105.1 ^b^	105.1	51.0 ^b^	51.0	-	-	-	-				
60	-	-	-	-	116.0 ^c^	116.1	135.3 ^c^	135.3				
P	0.02	0.29	0.001	0.27	<0.0001	0.13	<0.0001	0.33				
F value	3.80	1.46	8.05	1.57	33.67	2.55	36.45	1.32				

DAE is the number of days after plant emergence inoculum was applied to the potting soil.

AUDPC^i^ is a modified measure such that all treatments were assessed for the same period after initial inoculation treatment. The data was truncated back to ensure the same number of data points (three) was analysed for each curve.

Different superscripts denote significant differences (p < 0.05) within same columns using Tukey’s method.

Where disease progress was assessed from the date of inoculum addition and data truncated to three data points after inoculation (AUDPC^i^, Table 1) there was no significant differences (p > 0.05) identified in any of the three PTs indicating that the rate of increase in pathogen over this period was consistent across all treatments.

#### Root and tuber disease

**Fig 2:**
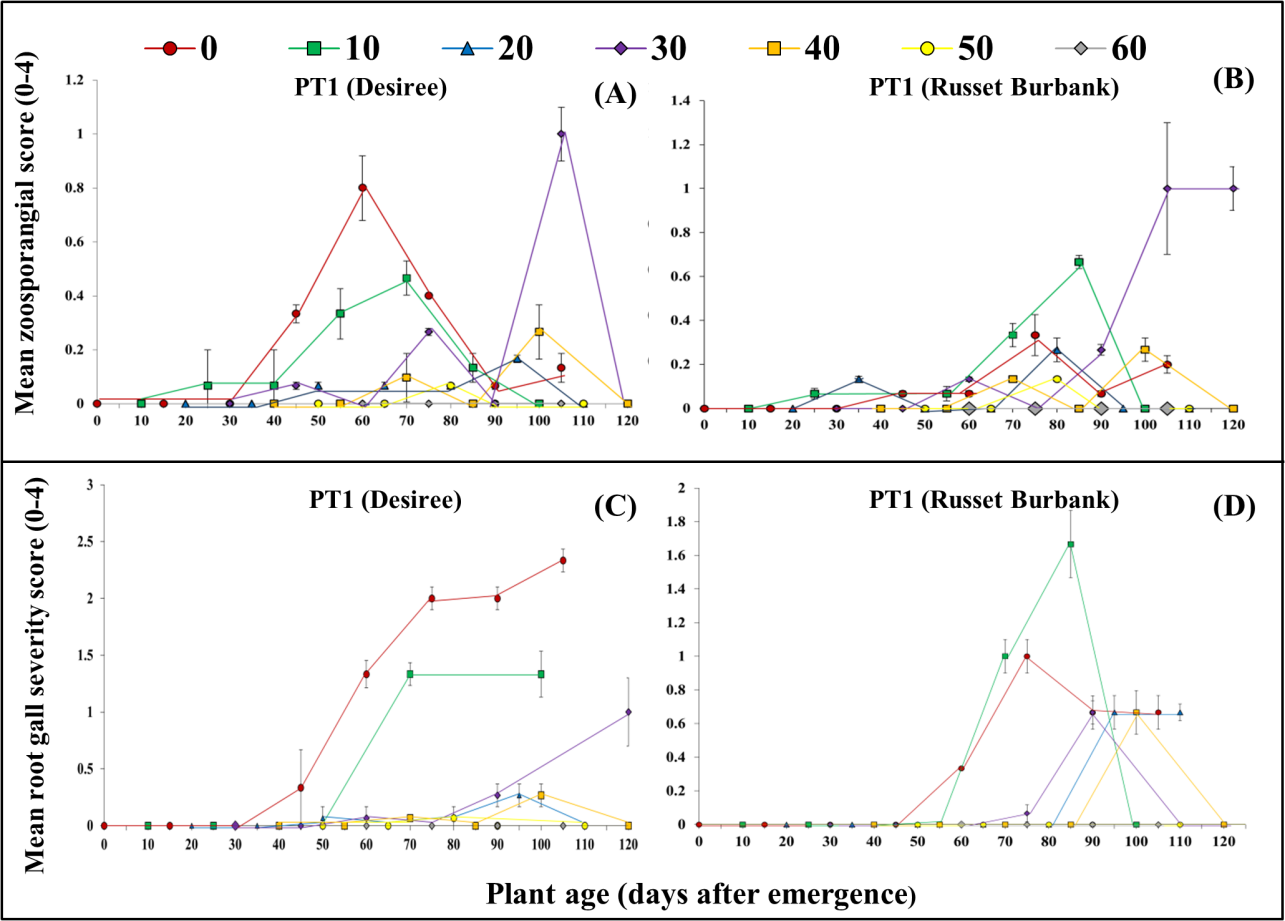
The effect of delayed inoculation of *S*. *subterranea* on presence of zoosporangia and galls in roots. Results are for two potato cultivars in pot trial 1 (winter) with A and B showing mean zoosporangial score (0–4) and C and D showing root gall severity score (0–4). Markers represent the different inoculation treatment dates shown as days after emergence (DAE). Vertical bars are standard errors (n = 6).

**Fig 3:**
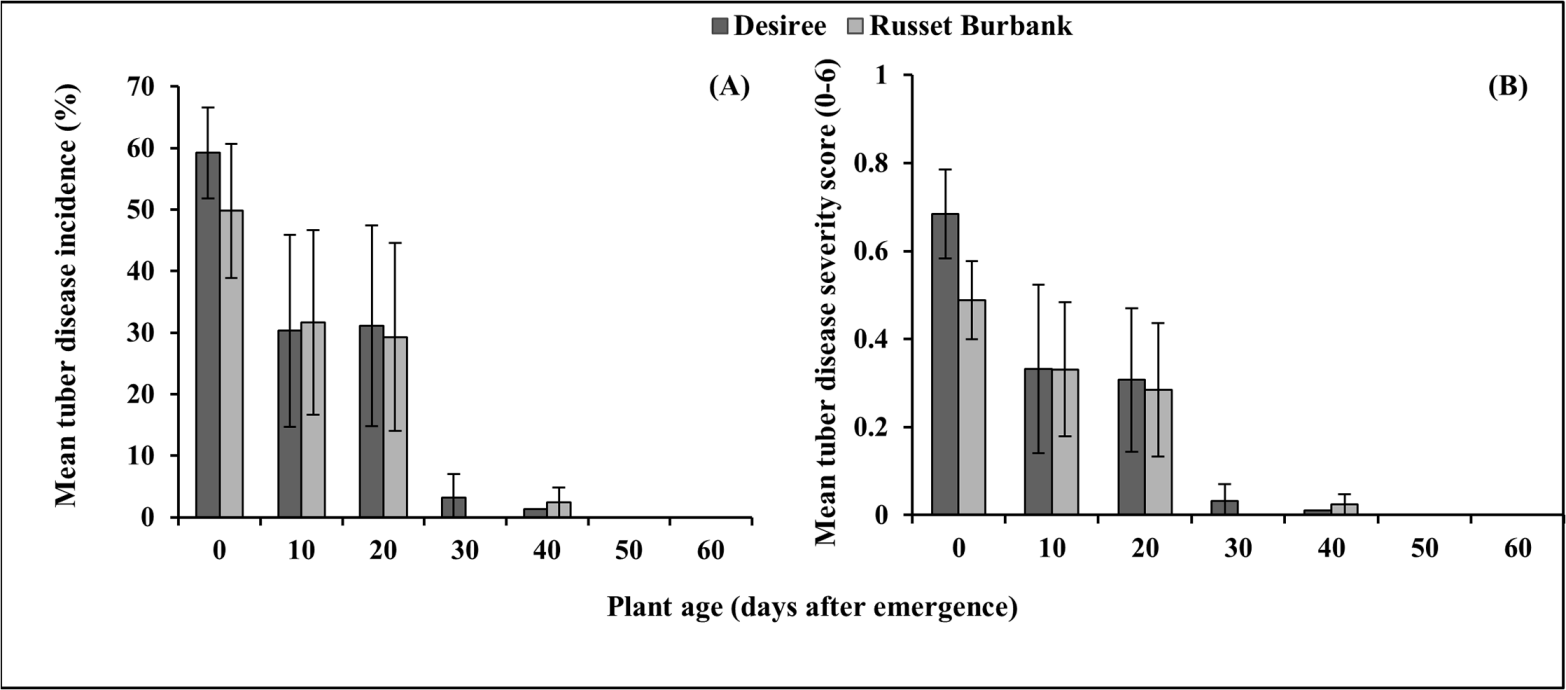
Effect of delayed inoculation of *S*. *subterranea* on tuber disease incidence (A) and severity (B). Results are for two potato cultivars in pot trial 1 (winter). Vertical bars are standard errors (n = 3).

In PT1, zoosporangia were observed within 15–45 days after inoculation in all inoculation date treatments except the 60 DAE treatment where no zoosporangia were observed in either cultivar (Fig 2A and 2B). There was a significant decrease in the AUDPC for the mean zoosporangia score following delayed inoculation in cultivar Desiree (p < 0.0001, Table 1A) with the 50 and 60 DAE treatments having less infection than the 0 and 10 DAE treatments. Whilst similar trends were seen in Russet Burbank the effects were not significant (p = 0.06, Table 1A). Significant decreases in the AUDPC for mean root gall score was found in both cultivars, Desiree (p = 0.003, Fig 2C) and Russet Burbank (p = 0.01, Fig 2D), in response to delayed inoculation. In Desiree, pathogen inoculation at emergence, 10, 20 and 30 DAE led to a significantly greater root gall score than the inoculation at 60 DAE. In Russet Burbank the inoculation at 10 DAE produced significantly greater gall score than the 60 DAE inoculation treatment (Table 1A). In PT1, where the analysis accounted for the altered application dates with data truncated to three data points after inoculation (AUDPC^i^, Table 1A) there was no significant differences (p > 0.05) identified for either zoosporangial score or galling indicating that these parameters was consistent across all treatments. In PT1, there were also significant decreases in mean tuber disease incidence (p < 0.001, Fig 3A) and severity (p < 0.001, Fig 3B) following delaying inoculation in both cultivars. Inoculation at emergence, 10 and 20 DAE had significantly greater disease incidence and severity than at 30, 40, 50 and 60 DAE.

In PT2 and PT3 sporadic occurrence of zoosporangia were observed but not scored. No root galling was found in plants from either trial and no disease was present in tubers harvested from plants in PT2.

### Impact of seed and soil chemical treatment on *Spongospora* infection and powdery scab

#### Pot trials

**Fig 4:**
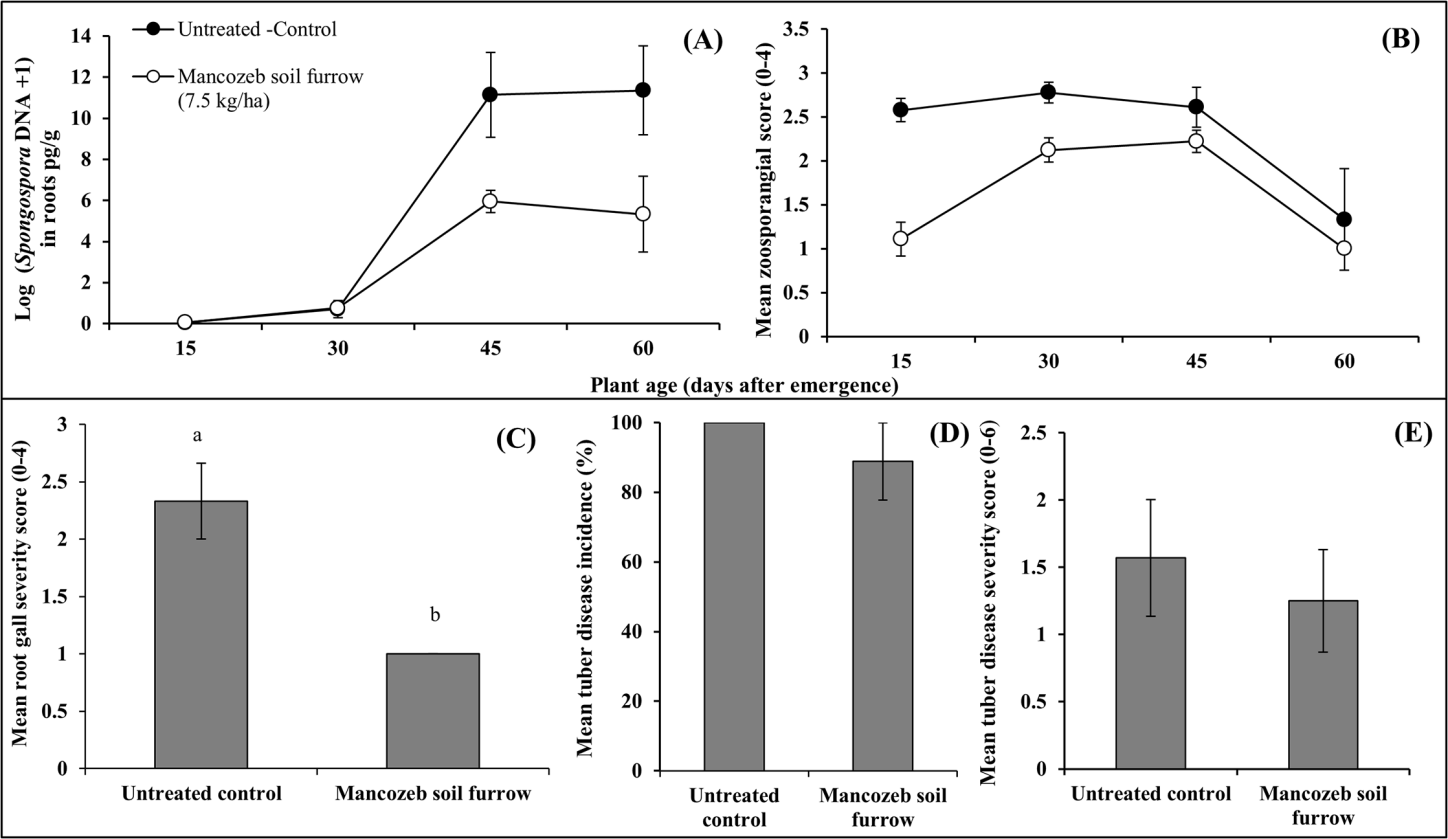
The impact of mancozeb soil furrow treatment (7.5kg/ha) on root infection and disease in cultivar Russet Burbank in a pot trial (PT4—summer). Multiple sequential assessments measured A- *S*. *subterranea* DNA concentration (pg/gm) in roots (n = 9); B- zoosporangia observation score (n = 3). Two assessments were made for C- root gall severity at 45 and 60 days (data pooled) after emergence (n = 3); and single assessments for D- mean tuber disease incidence and E- mean tuber disease severity at plant senescence (n = 3). Vertical bars are standard errors.

In PT4 the amount of *S*. *subterranea* DNA present in Russet Burbank potato roots increased in both fungicide treated and control treatment but the rate of increase was significantly less, over time, in the soil furrow mancozeb treatment (p < 0.001, Fig 4A, Table 2). Zoosporangial score was also reduced by mancozeb treatment, with the effect greater in the earlier assessments contributing to a significant reduction in AUDPC compared to the untreated control (p < 0.025, Fig 4B, Table 2). Similarly, root galling (pooled at 45 and 60 days) was significantly reduced by mancozeb treatment (p = 0.045, Fig 4C) while tuber disease incidence (Fig 4D) and severity (Fig 4E) showed non-significant reductions by the fungicide soil treatment.

**Table 2:** Area Under Disease Progress Curve for root pathogen and disease parameters following the seed tuber and soil fungicide treatment (PT 4 and 5; FT 1 and 2).

	PT4		PT5	FT1			FT2		
Treatment	Pathogen DNA	Zoosporangial score	Zoosporangial score	Pathogen DNA	Zoosporangial score	Root galling score	Pathogen DNA	Zoosporangial score	Root galling score
Untreated control	344.7^a^	64.2^a^	36.3^a^	393.3^a^	6.7^a^	48.8^a^	238.8	10.5^a^	54.4^a^
Formalin seed dip (1%)				205.6^b^	0.7^c^	13.1^b^	139.7	1.8^b^	9.4^b^
Fluazinam seed dip (7.5 g/10 kg seed)				180.4^bc^	2.2^bc^	11.3^b^	94.5	2.3^b^	16.9^b^
Mancozeb seed dip (32 g/10 kg seed)				115.3^bc^	2.2^bc^	15.0^b^	174.3	1.5^b^	18.8^b^
Fluazinam soil furrow (3.0 kg/ha)				257.8^b^	3.0^b^	0.0^c^	162.2	1.5^b^	18.8^b^
Mancozeb soil furrow (3.25 kg/ha)			12.8^b^						
Mancozeb soil furrow (7.5 kg/ha)	133.1^b^	32.2^b^	10.5^b^	368.9^a^	3.8^b^	56.3^a^	173.1	5.3^a^	43.8^a^
Mancozeb soil furrow (15 kg/ha)			9.5^b^						
P	<0.001	<0.025	0.02	<0.001	0.002	0.01	0.07	0.004	0.04
F value	9.24	3.71	3.91	12.60	5.39	3.66	2.35	7.65	3.21

Different superscripts denote significant differences (p < 0.05) within same columns using Tukey’s method.

**Fig 5:**
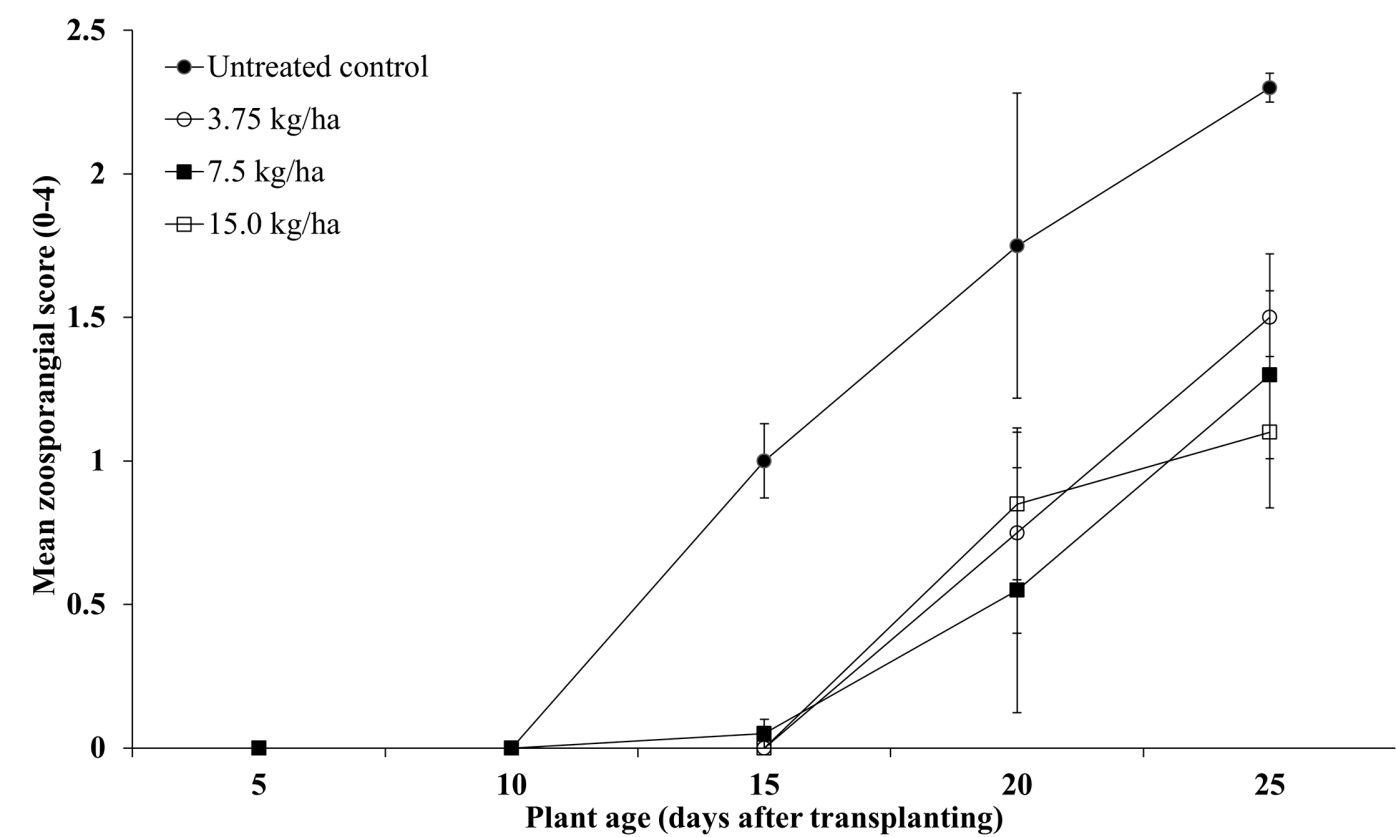
The effect of different mancozeb soil furrow treatments (0, 3.25, 7.5 or 15.0 kg/ha) on mean zoosporangial score (0–4) in potato roots grown in a pot trial (PT5 –autumn/winter). Data was pooled across the cvs. Desiree and Russet Burbank. Vertical bars are standard errors (n = 6).

In PT5, where only the zoosporangial score was measured for a 25 day period, there were no significant differences seen between Desiree and Russet Burbank (p > 0.05) for each assessment and these data were pooled to provide a larger data set for subsequent analyses. All mancozeb treatments (3.25kg /ha,7.5kg/ha, 15.0 kg/ha) significantly reduced the zoosporangial score and slowed the rate of infection compared to the untreated control (p <0.001, Fig 5, Table 2).

#### Field trials

Pre-plant soil inoculum levels were very low and seed inoculum levels very high in both field trials. Both trials produced high levels of *S*. *subterranea* root infection, root galling, and tuber disease in both cultivars. Exploratory analysis of the data indicated that there were no significant effects of cultivar on the disease outcomes assessed and thus data sets from Russet Burbank and Innovator were pooled to provide a larger data set.

**Fig 6:**
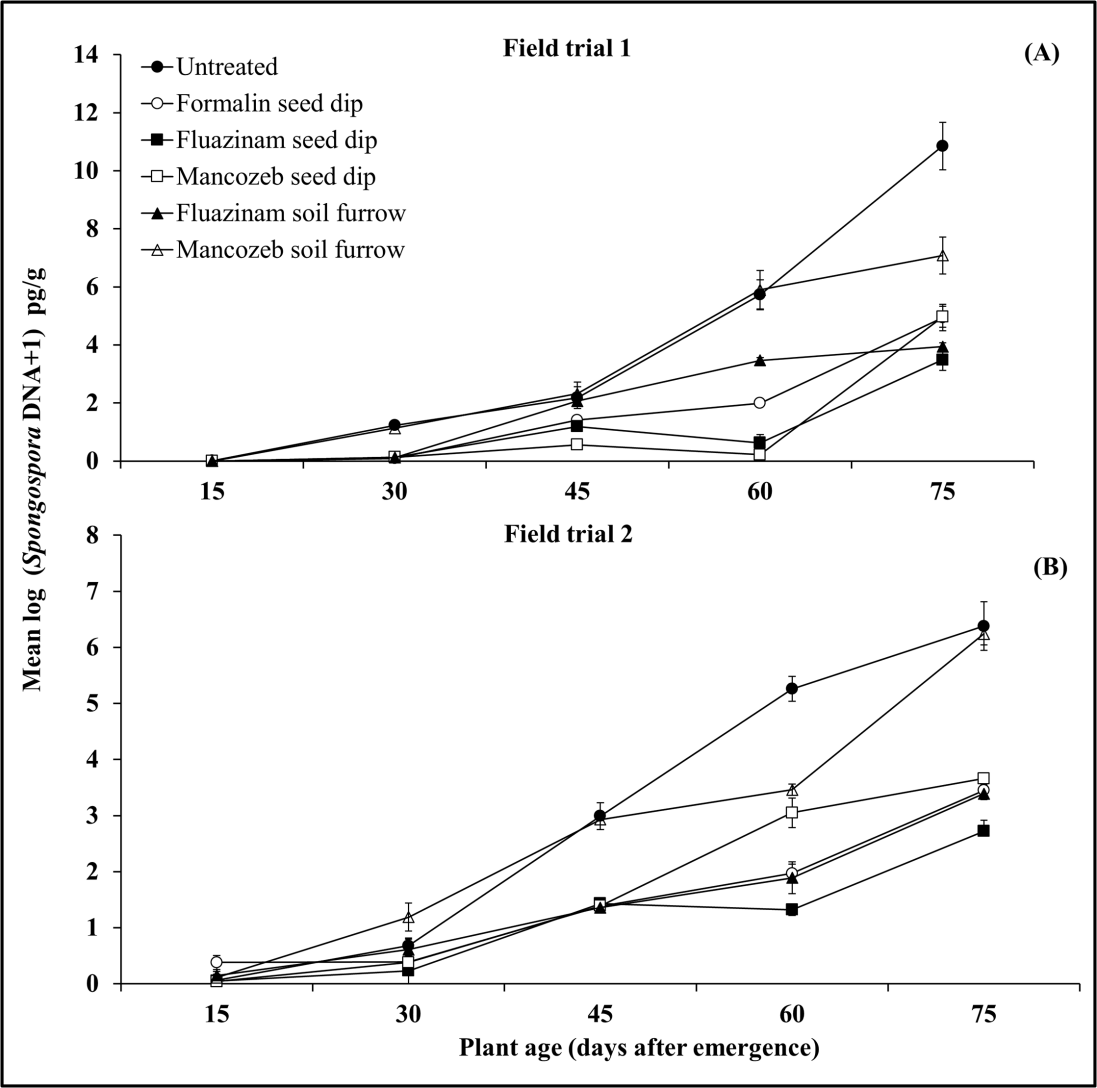
The effect of fungicide seed and soil treatments on the increase in pathogen content (pg *S*. *subterranea* DNA/gm) in potato roots. Measurements were made 15–75 days after emergence (DAE) in two field trials (A—FT1, B—FT2). Data was pooled across the cultivars Russet Burbank and Innovator, vertical bars are standard errors (n = 9).

The amount of *S*. *subterranea* DNA detected within roots increased with time over the five sequential assessment dates. The AUDPC analysis identified significant treatment differences in FT1 (p < 0.0001, Fig 6A, Table 2) but not in FT2 (p = 0.07, Fig 6B, Table 2). Essentially, the untreated control and mancozeb soil furrow treatments had significantly higher AUDPC’s than all other treatments in FT1, with analogous trends seen in FT2 indicating an increased rate of pathogen replication in these two treatments.

**Fig 7:**
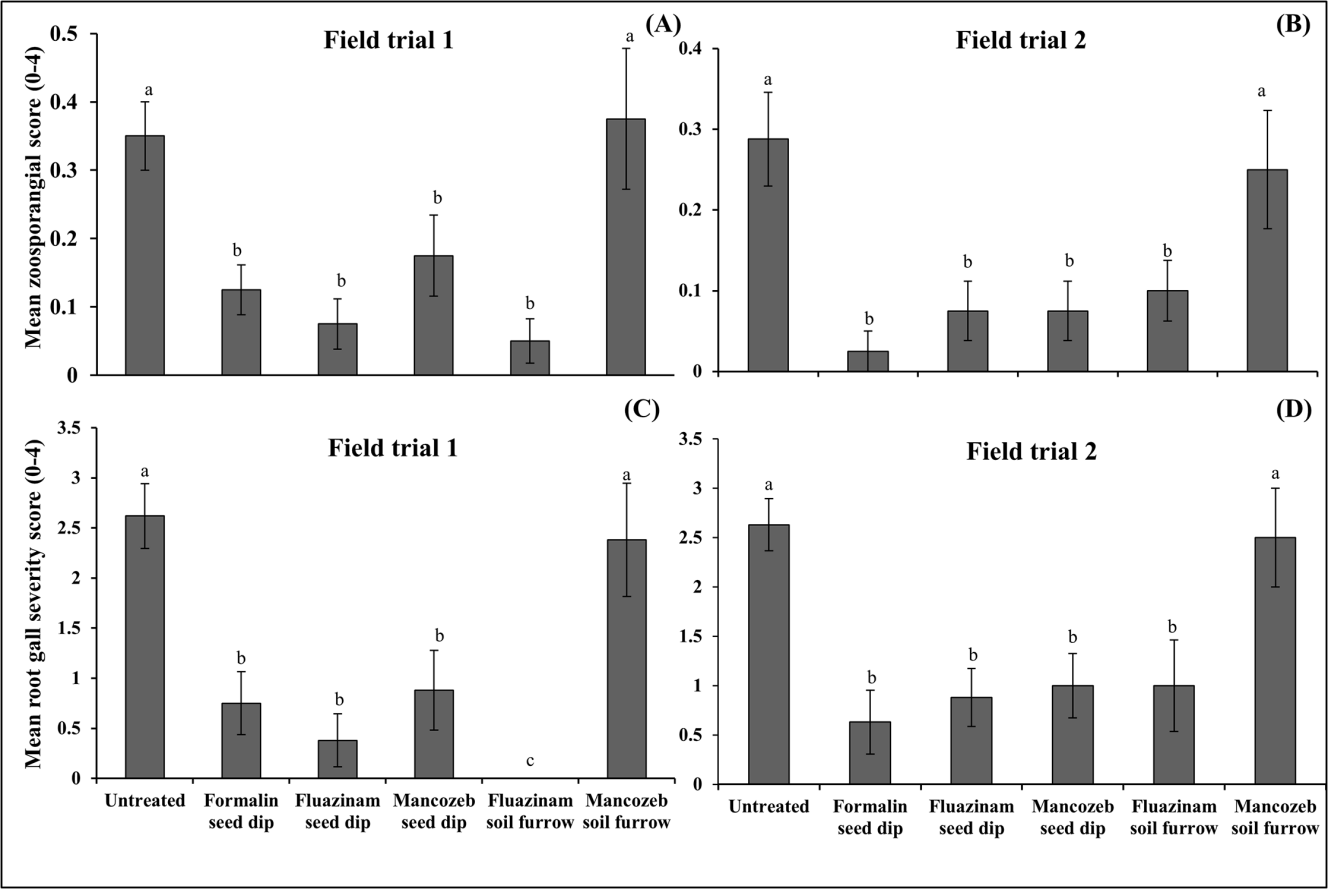
The effect of fungicide seed and soil treatments on root infection. Measurements included mean potato root zoosporangia infection score (0–4) in FT1 (A) and FT2 (B) and root gall severity score (0–4) in FT1 (C) and FT2 (D). Data was pooled across the cultivars Russet Burbank and Innovator and two assessment dates, 45 and 60 days after emergence. Vertical bars are standard errors (n = 8).

The soil treatment (fluazinam) and seed dips (formalin, fluazinam and mancozeb) consistently reduced (p < 0.05; 2–6 fold decrease, Fig 7A and 7B) mean zoosporangial score compared to the untreated control in both field trials. The mancozeb soil treatment produced a zoosporangial score equivalent (p > 0.05, Table 2) to the untreated control indicating that root infection developed more rapidly in these two treatments.

Root galls were first observed at 45 days after plant emergence with consistent root galling observed across all treatments at 60 and 75 days after emergence (galling data was pooled across these last two assessment dates for Fig 7C and 7D). Consistent with the root infection data, similar trends were observed with the soil treatment (fluazinam) and seed dips (formalin, fluazinam and mancozeb) reducing (p < 0.05; 2–8 fold decrease) root gall score and root gall production rate (AUDPC, Table 2) compared to the untreated control. Once again, the mancozeb soil treatment and the untreated control produced similar (p > 0.05) levels of moderately high galling (root gall scores of 2.3–2.6).

**Fig 8:**
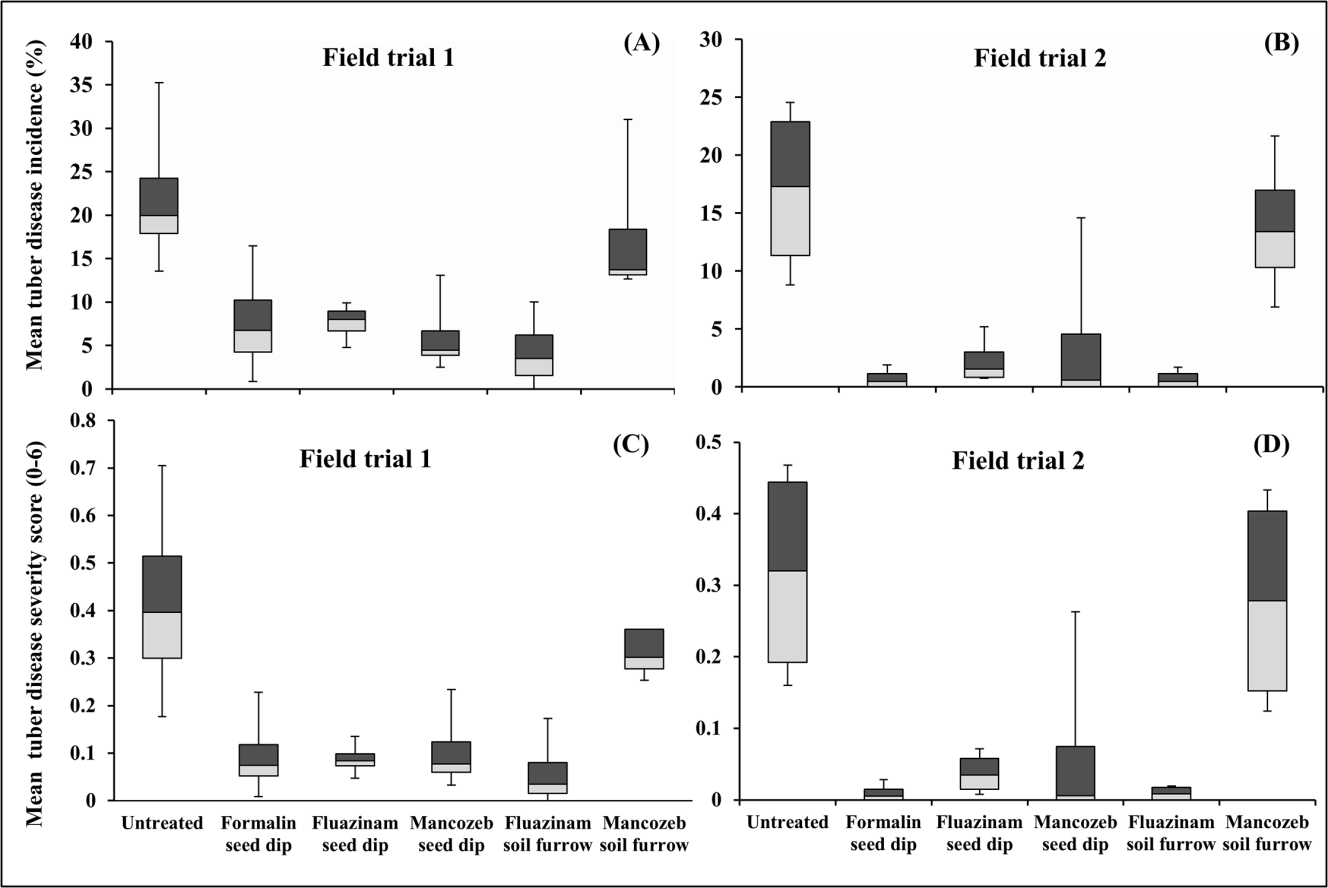
The effect of fungicide seed and soil treatments on mean tuber disease. Measurements included disease incidence in FT1 (A) and FT2 (B) and severity (0–6) in FT1 (C) and FT2 (D). Data was pooled across the cultivars Russet Burbank and Innovator. Vertical bars are the variation within the population (n = 8).

The soil treatment (fluazinam) and seed dips (formalin, fluazinam and mancozeb) also significantly reduced mean tuber disease incidence and severity in both trials (FT1 and FT2). In FT1, both disease incidence (p = 0.01, Fig 8A) and disease severity (p = 0.003, Fig 8C) were significantly lower in the soil furrow treatment (fluazinam) and seed dip treatments (fluazinam, formalin and mancozeb) than the untreated control and mancozeb soil furrow treatment. The same trend was found in FT2 for both disease incidence (p = 0.001, Fig 8B) and severity (p = 0.003, Fig 8D).

## Discussion

Most previous studies with *S*. *subterranea* have focused on root galling and tuber lesions that occur late in the infection cycle. Yet the most critical interaction between pathogen and plant occurs much earlier, following initiation of root infection and subsequent polycyclic development of further root infections rapidly building pathogen populations which subsequently lead to expression of visual disease [6,7,9,10]. Recently, the use of qPCR to detect and quantify pathogen content within roots has been used to differentiate cultivars on the basis of resistance to root infection [8]. Other studies have emphasized the dynamics of root infection in informing subsequent disease progress [9]. Work presented here highlights the benefits of monitoring the temporal patterns of pathogen development during infection, demonstrating that two distinct pathways (delayed infection and a reduced epidemic rate) can substantially suppress disease using delayed inoculation and seed and soil treatments as test cases.

### Impact of delayed inoculum application

In this study, plants receiving inoculum earlier built pathogen levels faster and produced greater root and tuber disease. *S subterranea* DNA was detected 15–20 days after inoculation with zoosporangia and root galls observed 15–45 and 45–75 days after inoculation respectively. Tuber disease required inoculation by 40 DAE to occur. Within the average potato life cycle (100–120 days), the expression of powdery scab tuber symptoms and root galls require 60 days [9,10]; this was evidenced in our study by altering the inoculation date. While both cultivars tested showed similar trends, the levels of infection, both in roots and tubers was greater in the moderately susceptible Desiree than the moderately resistant Russet Burbank [23]. From this study it is clear that any strategy that can delay the interaction between pathogen and host roots would be beneficial in reducing disease.

We also demonstrated that roots and tubers have different periods of susceptibility to pathogen infection. Roots are susceptible to *S*. *subterranea* throughout the whole plant growth cycle with infections initiating 15–20 days after inoculation and disease progressing in a rapid manner at a similar rate regardless of plant species (potato or tomato) or plant age at infection. Conversely, prior studies have shown tubers have a narrow window of susceptibility around early tuber initiation [9,10,11], with infections postulated to occur through immature lenticels [24,25,26]. Where infection can be restricted or delayed through this tuber development phase, tuber lesions can be reduced.

### Impact of seed and soil chemical treatments

Several fungicides have been tested for the control of powdery scab as soil or tuber treatments, with varying levels of efficacy [10,11,12]. High levels of elemental sulphur and Zinc-EDTA has been shown to reduce disease severity when applied to the soil but this has only limited efficacy [13]. Formalin has shown effective control but has been associated with poor plant emergence and growth [6,12]. Fluazinam and flusulfamide have shown promise as soil treatments, but the effects are not always consistent [3]. When testing a range of chemicals applied to the seed, foliage or furrow, Braithwaite and colleagues [12] identified alternatives including fluazinam, mancozeb, maneb, dichlorophen-Na for controlling powdery scab symptoms. These chemicals were able to significantly control powdery scab disease with reductions of 66%-95% recorded, although no treatment provided complete control. All of these aforementioned studies focused on tuber symptom development in field and pot trials with plants grown to full maturity. The sometimes erratic nature of the disease and uneven pathogen distribution within field sites can lead to inconsistent results when screening possible new chemical treatments [9,27]. Even in our studies, visual expression of root and tuber disease was not always found. However, tracking pathogen development within the root system proved to be highly reliable and provided an understanding of the efficacy and activity of these fungicide treatment on both root and tuber infection.

Consistent with previous work [6,12] fluazinam, mancozeb and formalin dips along with fluazinam soil furrow treatment was able to reduce root galling and tuber disease. Importantly we have identified the impact of these treatments on initiation of root infection and pathogen development. In contrast to the delayed inoculation experiments, effective seed and soil-applied fungicide treatments (with the possible exception of some treatments in FT1) did not delay onset of infection but rather slowed pathogen development resulting in less disease. This early identification of treatment efficacy can provide a rapid means of screening and identifying fungicides effective against this disease.

The mancozeb soil treatment gave good control in pot trials but failed to suppress pathogen development in roots and tubers in the field trials. Similar erratic responses of this fungicide applied as a soil treatment have been seen (F. Mulcahy, Simplot Australia Pty Ltd, pers. comm.). The contrasting results in our trials may relate to the major inoculum sources used. In the pot trials, a high dose rate of soil inoculum was the sole source of pathogen. In the field trials despite presence of disease in previous potato crops on these sites, the soil tests prior to planting indicated low soil inoculum levels. The major inoculum source for these trials was rather from the infested seed tubers. With the unavailability of fluazinam as a registered fungicide for potato within Australia there is a need to assess other means of control which may form an integrated component of managing this disease [12,13].

Root galling and tuber disease is influenced by soil environmental conditions [6,23] at the critical period of tuber susceptibility [25,28]. Tuber disease is favoured by cool soil temperatures within the range of 9–17°C whilst root galling occurs within a warmer soil temperature range of 11–25°C) [9,29]. Plants in PT2 and PT3 failed to produce root galling and in PT2 tuber lesions. We can speculate that the high summer temperatures regularly experienced in the glasshouse over the trial period may have inhibited root galling and tuber disease expression. However, *S*. *subterranea* was successfully quantified within the roots of both trials and treatments were able to be compared for efficacy in reducing pathogen development highlighting the reliability benefits of this assay.

Tracking disease progress curves of *S*. *subterranea* within potato roots as we have done here has proven to be valuable in furthering our understanding of the epidemiology of this disease and in providing a reliable tool for rapid assessment of diverse disease mitigation strategies. The critical role of root infection in disease expression is clear, and we show both delaying infection and slowing epidemic rate provide substantial disease control.
